# Dr. I. C. St. John

**Published:** 1899-06

**Authors:** 


### OBITUARY.

### Dr. I. C. St. John.

Died June 3rd, at the home of his parents, Winona, Wisconsin, June 3rd, at 10:30 A. M., Dr. I. C. St. John.

The doctor had been in feeble health for some two or three months, but it was not thought that he was dangerously ill until very near the last. There seemed to be a difficulty with his heart and the circulation. He was able to attend to his practice till within a few days of his decease. Dr. St. John has been a successful practitioner of dentistry in Minneapolis for the last sixteen years. He was one of the most prominent dentists of that city, and was regarded as authority on many things in his profession. He was a graduate from the University of Michigan in 1880 and has confined himself strictly to the practice of his profession all these years, probably more closely than his physical ability could well bear. He was so attached however to his profession, that he was most happy when most fully engaged in it. He was a warm and most unselfish friend, and upright in his life, one who had little or no patience with shams or false pretense.

When such as he pass away it leaves a gap not easily filled. Those of his home, and who are so fortunate as to fully know him, will have the deep sympathy of all.

